# Src Family Kinases Facilitate the Crosstalk between CGRP and Cytokines in Sensitizing Trigeminal Ganglion via Transmitting CGRP Receptor/PKA Pathway

**DOI:** 10.3390/cells11213498

**Published:** 2022-11-04

**Authors:** Lingdi Nie, Kai Sun, Ziyang Gong, Haoyang Li, John P. Quinn, Minyan Wang

**Affiliations:** 1 Centre for Neuroscience, Department of Biological Sciences, Xi’an Jiaotong-Liverpool University (XJTLU), Suzhou 215123, China; 2 Department of Pharmacology and Therapeutics, Institute of Systems, Molecular and Integrative Biology, Liverpool L69 7ZB, UK

**Keywords:** Src family kinases, calcitonin gene-related peptide, interleukin-1β, C-C motif ligand 2, C-X-C motif ligand 1, protein kinase A, trigeminal ganglion, migraine

## Abstract

The communication between calcitonin gene-related peptide (CGRP) and cytokines plays a prominent role in maintaining trigeminal ganglion (TG) and trigeminovascular sensitization. However, the underlying regulatory mechanism is elusive. In this study, we explored the hypothesis that Src family kinases (SFKs) activity facilitates the crosstalk between CGRP and cytokines in sensitizing TG. Mouse TG tissue culture was performed to study CGRP release by enzyme-linked immunosorbent assay, cytokine release by multiplex assay, cytokine gene expression by quantitative polymerase chain reaction, and phosphorylated SFKs level by western blot. The results demonstrated that a SFKs activator, pYEEI (YGRKKRRQRRREPQY(PO3H2)EEIPIYL) alone, did not alter CGRP release or the inflammatory cytokine interleukin-1β (IL-1β) gene expression in the mouse TG. In contrast, a SFKs inhibitor, saracatinib, restored CGRP release, the inflammatory cytokines IL-1β, C-X-C motif ligand 1, C-C motif ligand 2 (CCL2) release, and IL-1β, CCL2 gene expression when the mouse TG was pre-sensitized with hydrogen peroxide and CGRP respectively. Consistently with this, the phosphorylated SFKs level was increased by both hydrogen peroxide and CGRP in the mouse TG, which was reduced by a CGRP receptor inhibitor BIBN4096 and a protein kinase A (PKA) inhibitor PKI (14–22) Amide. The present study demonstrates that SFKs activity plays a pivotal role in facilitating the crosstalk between CGRP and cytokines by transmitting CGRP receptor/PKA signaling to potentiate TG sensitization and ultimately trigeminovascular sensitization.

## 1. Introduction

Migraine is a recurrent primary headache disorder that afflicts approximately 15% of the population worldwide [1]. A key mechanism by which nearly all migraine triggers induce migraine attacks is the activation and sensitization of the trigeminovascular pathway [2,3,4]. As an important peripheral component of the trigeminovascular pathway, trigeminal ganglion (TG) contains the cell bodies of meningeal nociceptors, the activation of which initiate trigeminovascular activation [5,6,7]. Active signaling mediated mainly by neuropeptides and inflammatory mediators occurs within the TG, among which calcitonin gene-related peptide (CGRP), the key drug target of migraine prevention and therapy, is a key player [8]. In the TG, released CGRP binds to CGRP receptor to facilitate neuronal excitability [9,10,11] and neuroinflammation, including elevated release and expression of inflammatory cytokines [11,12,13,14]. Importantly, cytokines can signal back to neurons, which promotes CGRP synthesis and release [15,16], thereby inducing a positive feedback loop of sensitization. Thus, the communication between CGRP and cytokines plays a prominent role in maintaining TG activation and sensitization as well as trigeminovascular sensitization [17,18,19], although the underlying regulatory mechanism is elusive.

Src family kinases (SFKs) activity has been previously found to mediate CGRP release in dorsal root ganglion neurons [20] and TG [21]. SFKs activity also mediates inflammatory cytokine release and expression in primary glial cells [22,23,24,25] and mediates interleukin-1 β (IL-1β) gene expression in the mouse TG [21]. Importantly, SFKs are known to play a key role in migraine pathogenesis. In an inflammatory soup-induced chronic migraine model, central inhibition of SFKs attenuates mechanical allodynia and synaptic plasticity [26]. In a genetic mouse migraine with aura model familial hemiplegic migraine type 2 (FHM2), deactivation of SFKs reduces the Ca^2+^ sensitivity and contraction of the cerebral arteries, which contributes to vascular tone and brain perfusion abnormalities [27]. Similarly, systemic deactivation of SFKs reduces cortical spreading depression (CSD), a migraine with aura model, and CSD-induced cerebral cortical inflammatory cytokines interleukin 1 beta (IL-1β) and tumor necrosis factor alpha (TNFα) gene expression [28]. Taken together, it is likely that SFKs activity facilitates the communication between CGRP and cytokines to activate and sensitize TG, which requires clarification.

In the present study, we examined whether SFKs activity facilitates the crosstalk between CGRP release and cytokines release and gene expression to activate and sensitize the mouse TG. How SFKs activity mediates the communication between CGRP and cytokines in TG is also explored by investigating the involvement of CGRP receptor/protein kinase A (PKA) pathway.

## 2. Materials and Methods

### 2.1. Animals

A total of 143 adult male C57BL/6J mice (21.4 ± 0.17 g) were used and purchased from Shanghai SLAC Laboratory Animal Corporation Ltd. (Shanghai, China). All studies in this paper were carried out in male rodents so that the effect of hormonal fluctuation in females is minimized. Mice were housed in the Experimental Animal Centre of Soochow University for at least one week to be acclimated to the housing room before use. Animal procedures were approved by the Ethical Review Panels of Xi’an Jiaotong–Liverpool University (XJTLU) under the agreement with Soochow University and performed in accordance with relevant China national and provincial guidelines. For each experiment, randomization of experimental groups was performed to reduce bias. All animals used were randomly allocated to different experimental groups.

### 2.2. Mouse TG Tissue Culture

Isolated TG culture is a commonly used model to study TG molecular and neurophysiological properties. Signaling molecules produced in TG cell bodies are delivered to the peripheral and central terminals via axonal transport to give rise to sensory transduction and neurotransmission [6,29,30,31]. Therefore, isolated TG culture is commonly used as a model of its peripheral or central endings to study meningeal nociceptors and trigeminal nociceptive transmission [17,19,32]. The method of TG tissue culture was established as reported previously [21]. Mice were sacrificed by rapid cervical dislocation. Both the left and right TG of each mouse were collected, and the merged TG were used for one individual experiment. The TG were recovered in 300 μL pre-oxygenated Kreb’s solution (composition in mM: 126 NaCl, 2.5 KCl, 2.4 CaCl_2_·2H_2_O, 1.3 MgCl_2_·6H_2_O, 18 NaHCO_3_, 1.2 NaH_2_PO_4_, 10 glucose; pH 7.4) for 30 min at 37 °C and then washed with pre-oxygenated Kreb’s solution every 5 min for 30 min. Subsequently, the TG were incubated with each drug for 20 min or 1 h at 37 °C.

In order to explore whether SFKs activity mediates the communication between CGRP and cytokines in TG, three series of experiments were designed. Series 1: whether activation of SFKs increases CGRP release and IL-1β gene expression was examined in cultured mouse TG. This model has been validated in our previous publication by using KCl, a known trigger for neuronal activation and CGRP release, which successfully induces CGRP release [21]. A SFKs activator, pYEEI (YGRKKRRQRRREPQY(PO3H2)EEIPIYL) [33,34], or its negative control, the non-phosphorylated peptide, YEEI (YGRKKRRQRRREPQYEEIPIYL), was applied at 1 mM [35,36] for 20 min. pYEEI binds to the SH2 domains of SFKs which hampers their closing conformation at the inactive state and induces their open active conformation [34,37,38]. A cell-penetrating peptide TAT (YGRKKRRQRRR) [39] was conjugated to both pYEEI and YEEI to make them cell permeable. These peptides were customized by A^+^ Peptide (Shanghai, China). Two groups were designed: (i) 1 mM pYEEI; (ii) 1 mM YEEI (*n* = 8 for each). The level of CGRP released into the culture medium was detected by enzyme-linked immunosorbent assay (ELISA); the mRNA level of IL-1β in the TG was detected by quantitative polymerase chain reaction (qPCR). Series 2: whether deactivation of SFKs reduces CGRP release was examined in cultured mouse TG pre-sensitized by reactive oxygen species (ROS). Stress is the top trigger for migraineurs [40], and ROS is a common trigger of oxidative stress [41]. Since hydrogen peroxide (H_2_O_2_), a type of ROS, can induce CGRP release in dorsal root ganglion neurons [42], the present study used H_2_O_2_ to induce CGRP release in the TG. To inhibit SFKs activity, a SFKs inhibitor saracatinib (S1006, Selleckchem, Houston, TX, USA), which binds to the kinase (SH1) domains of SFKs [43], was used because saracatinib has been tested for treating different types of cancer [44,45,46] and Alzheimer’s disease [47,48] in clinical trials and showed good tolerability and safety in patients. The cultured TG was treated with Kreb’s, 1 mM H_2_O_2_ [42], 1.5 µM, 4 µM, or 10 µM saracatinib [49,50] in the presence of 1 mM H_2_O_2_ for 20 min. For this series, five groups were designed: (i) Kreb’s (*n* = 8); (ii) 1 mM H_2_O_2_ (*n* = 8); (iii) 1 mM H_2_O_2_ + 1.5 µM saracatinib (*n* = 8); (iv) 1 mM H_2_O_2_ + 4 µM saracatinib (*n* = 7); (v) 1 mM H_2_O_2_ + 10 µM saracatinib (*n* = 7). Series 3: whether deactivation of SFKs reduces the release and gene expression of inflammatory cytokines induced by CGRP was examined in cultured mouse TG. CGRP (SCNTATCVTHRLAGLLSRSGGVVKDNFVPTNVGSEAF-NH2, disulfide bridge: Cys2-Cys7, A^+^ Peptide, Shanghai, China), the potent neuroinflammatory mediator [51], was used to induce the release and gene expression of inflammatory cytokines in the TG, and the effect of the SFKs inhibitor saracatinib (S1006, Selleckchem, Houston, TX, USA) on these phenomena was studied. For studying cytokine release, the cultured TG was treated with Kreb’s, 3 µM CGRP [9], or 1.5 µM saracatinib [49,50] in the presence of 3 µM CGRP for 20 min; for studying cytokine gene expression, the treatments were the same except for that 4 µM saracatinib in the presence of 3 µM CGRP was applied as an additional group, and the treatment time for each group was 60 min. Four groups were therefore designed: (i) Kreb’s; (ii) 3 µM CGRP; (iii) 3 µM CGRP + 1.5 µM saracatinib; (iv) 3 µM CGRP + 4 µM saracatinib (*n* = 8 for each). To detect the release of multiple cytokines into the culture medium, a multiplex immunoassay was used to measure the levels of 12 pro-inflammatory cytokines, C-C motif ligand 2 (CCL2), C-C motif ligand 5 (CCL5), C-X-C motif ligand 1 (CXCL1), C-X-C motif ligand 10 (CXCL10), granulocyte-macrophage colony-stimulating factor (GM-CSF), interferon alpha (IFN-α), interferon beta (IFN-β), interferon gamma (IFN-γ), IL-1β, interleukin 6 (IL-6), interleukin 12 (IL-12), TNFα, and one anti-inflammatory cytokine, interleukin 10 (IL-10). Only the cytokines whose levels in the TG culture medium were significantly altered by both CGRP and saracatinib were selected to detect their mRNA levels by qPCR.

In order to explore how SFKs activity mediates the communication between CGRP and cytokines in TG, the signaling pathway that SFKs transmit during these processes is investigated, for which three series of experiments were designed. Series 4: to ensure that SFKs activity is increased by H_2_O_2_ in the mouse TG in Series 2, the TG treated with Kreb’s and 1 mM H_2_O_2_ were collected to measure SFKs activity represented by the level of phosphorylated SFKs at Y416 using western blot in order to minimize animal use. How SFKs activity is enhanced by H_2_O_2_ was then investigated by examining whether inhibition of CGRP receptor reduces H_2_O_2_-enhanced SFKs activity in cultured mouse TG. To inhibit CGRP receptor, a CGRP receptor inhibitor, BIBN4096 (4561, Tocris, Bristol, UK), was used. The cultured TG was treated with 10 µM BIBN4096 in the presence of 1 mM H_2_O_2_ for 20 min. One additional group was designed: 1 mM H_2_O_2_ + 10 µM BIBN4096 (*n* = 7). Series 5: to ensure that SFKs activity is increased by CGRP in the mouse TG in Series 3, the TG treated with Kreb’s and 3 µM CGRP for 20 min were collected to measure the level of phosphorylated SFKs at Y416 using Western blot. Next, how SFKs activity is enhanced by CGRP was investigated by examining whether SFKs activity transmits CGRP receptor/PKA pathway as PKA is known to transmit signaling downstream CGRP [52,53,54] and activate SFKs in several models [36,55,56]. Specifically, whether inhibition of CGRP receptor and deactivation of PKA reduce CGRP-enhanced SFKs activity was examined in cultured mouse TG. To deactivate PKA, a PKA inhibitor, PKI (14-22) Amide (476485, Sigma-Aldrich, St. Louis, MO, USA), was used. The cultured TG was treated with 3 µM BIBN4096 [57] or 30 µM PKI (14-22) Amide [58] in the presence of 3 µM CGRP for 20 min. Two additional groups were designed: 3 µM CGRP + 3 µM BIBN4096, 3 µM CGRP + 30 µM PKI (14-22) Amide (*n* = 8 for each). Series 6: whether SFKs co-localize with CGRP or receptor activity modifying protein 1 (RAMP1), the unique and essential functional CGRP receptor subunit [59,60], was also examined in mouse TG using immunohistochemistry.

### 2.3. ELISA

After TG tissue culture, the level of CGRP released into the culture medium was measured using a mouse CGRP ELISA kit (CSB-EQ027706MO, CUSABIO, Houston, TX, USA). Briefly, 100 μL medium and each of 8 serially diluted standard solutions were added into an assay plate pre-coated with CGRP antibody, which was then incubated at 37 °C for 2 h. Next, after removing the remaining liquid in the wells, 100 μL 1 × biotin-conjugated antibody specific for CGRP was added to each well followed by incubating at 37 °C for 1 h. The wells were then aspirated and washed, after which each well was added with 100 μL 1 × avidin conjugated horseradish peroxidase (HRP) and incubated at 37 °C for 1 h. Following further wash to remove any unbound substances, each well was added with 90 μL TMB substrate and incubated at 37 °C for 30 min in the dark. The reaction was stopped by adding 50 μL stop solution to each well and the OD of the wells was read at 450 nm, 540 nm, and 570 nm using a colorimetric microplate reader (BioTek, Winooski, VT, USA). The mean reading at 540 nm and 570 nm were subtracted from that at 450 nm, which corrected for optical imperfections. A standard curve relating the OD values to the concentration of CGRP (pg/mL) in the standard solutions was plotted and an equation of the curve was obtained. The OD values of the media were used to calculate their CGRP concentration (pg/mL) using the equation.

### 2.4. Multiplex Immunoassay

A multi-analyte flow assay kit (740621, Biolegend, San Diego, CA, USA) was used to detect the release of 12 pro-inflammatory cytokines, CCL2, CCL5, CXCL1, CXCL10, GM-CSF, IFN-α, IFN-β, IFN-γ, IL-1β, IL-6, IL-12, TNFα, and one anti-inflammatory cytokine—IL-10—into the TG culture medium. First, 25 μL medium and each of 8 serially diluted standard solutions were added into an assay plate followed by adding 25 μL assay buffer and 25 μL mixed beads, which was then shaken at 500 rpm at room temperature for 2 h in the dark. After aspirating and washing the plate, 25 μL detection antibodies was added into the plate followed by shaking at 500 rpm at room temperature for 1 h. Next, 25 μL streptavidin-phycoerythrin (SA-PE) was added, and the plate was shaken at 500 rpm at room temperature for 30 min. After aspirating and washing the plate again, the beads in the plate were resuspended, and the plate was read on CytoFLEX S Flow Cytometer (C01161, Beckman Coulter, Brea, CA, USA). Each of the mixed beads was conjugated with a type of allophycocyanin (APC) fluorescence and an antibody specific to one of the 13 cytokines so that each cytokine in the medium was captured by its specific bead. The APC fluorescence conjugated to each bead had a differing level, which could be recognized by the flow cytometer at 660 nm to distinguish among different beads and identify the corresponding cytokine of each bead. The SA-PE bound to the detection antibody provided fluorescent signal in proportion to the amount of a certain cytokine bound to each bead, which was read as PE signal fluorescence intensity by the flow cytometer at 585 nm. Using LEGENDplexTM Data Analysis Software 8.0 (Biolegend, San Diego, CA, USA), a standard curve relating the PE signal fluorescence intensities to the concentration of each of the 13 cytokines (pg/mL) in the standard solutions was plotted and an equation of the curve was obtained. The PE signal fluorescence intensities of the media were used to calculate the concentration (pg/mL) of each of the 13 cytokines using the equation.

### 2.5. qPCR

After 60 min of TG tissue culture, total RNA of mouse TG was extracted using TRIZOL reagent (T9424 Sigma-Aldrich, St. Louis, MO, USA) and was reverse transcribed to cDNA by a GoScript Reverse Transcription System (A5001 Promega, Madison, WI, USA). The mRNA levels of specific genes were detected by qPCR using GoTaq qPCR Master Mix (A6002, Promega, Madison, WI, USA). The qPCR reaction was performed in QuantStudio 5 Real-Time PCR System (Applied Biosystems, Waltham, MA, USA) under the following thermal cycling conditions: 95 °C for 2 min, 95 °C for 15 s, 60 °C for 1 min, and 60–95 °C for 1 min. The mRNA level of each target gene was presented as relative fold change by normalizing the individual mRNA level of the gene to the geometric mean of the mRNA levels of two housekeeping genes, β-actin, and peptidylprolyl isomerase A (PPIA). Primers specific to the target genes were shown as follows: IL-1β forward 5′ACTACAGGCTCCGAGATGAACAAC3′, reverse 5′CCCAAGGCCACAGGTATTTT3′; CCL2 forward 5′CACTCACCTGCTGCTACTCA3′, reverse 5′ GCTTGGTGACAAAAACTACAGC3′; ACTB forward 5′CTGTCCACCTTCCAGCAGAT3′, reverse 5′CGCAGCTCAGTAACAGTCCG3′; PPIA forward, 5′TTGCTGCAGACATGGTCAAC3′, reverse 5′TGTCTGCAAACAGCTCGAAG3′.

### 2.6. Western Blot

Total protein of mouse TG was extracted using sodium dodecyl sulfate (SDS, 74255, Sigma-Aldrich, St. Louis, MO, USA), as described previously [21]. The concentration of the extracted protein was measured using Bicinchoninic Acid Protein Assay Kit (P0010, Beyotime, Shanghai, China). The protein levels of phosphorylated SFKs at Y416 and SFKs were analyzed by Western blot. Except for that, the protein level of β-actin was also analyzed, which was used as an internal control to calculate the relative expression levels of phosphorylated SFKs at Y416 and SFKs. Protein samples were denatured with SDS polyacrylamide (SDS-PAGE) sample loading buffer (P0015, Beyotime, Shanghai, China) at 100 °C for 5 min. The protein samples were separated on a 10% SDS-PAGE gel followed by transfer onto nitrocellulose membranes (66485, Pall, Pensacola, FL, USA). The membranes were incubated in 5% milk at room temperature for 1 h, followed by incubation with anti-phospho-Y416 SFKs antibody (1:200, 6943, CST, Beverly, MA, USA) and anti-β-actin antibody (1:2000, 4970, CST, Beverly, MA, USA) at 4 °C overnight. Subsequently, the membranes were incubated with IRDye 680RD donkey anti-rabbit secondary antibody (1:5000, 925-68073, LI-COR, Lincoln, NE, USA) for 1 h in the dark. Odyssey Near-Infrared Fluorescent Imaging System (LI-COR, Lincoln, NE, USA) was used to detect the protein levels of phosphorylated SFKs at Y416 and β-actin on the membranes by scanning fluorescent signals at 700 nm. Next, the anti-phospho-Y416 SFK antibody on the membranes was stripped off using 0.2 M NaOH (134070010, Acros Organics, Geel, Belgium) for 15 min. After incubating in 5% milk, the membranes were incubated with anti-SFK antibody (1:1000, 2109, CST) at 4 °C overnight followed by incubating with the anti-rabbit secondary antibody and imaging to detect the protein level of SFKs. The mean gray value of protein band intensity was quantified using Image Studio Lite 5.0 (LI-COR, Lincoln, NE, USA). The level of phosphorylated SFKs at Y416 was presented as absolute ratio in the band intensities between phosphorylated SFKs at Y416 and β-actin, phosphorylated SFKs at Y416 and SFKs, and SFKs and β-actin.

### 2.7. Immunohistochemistry

As CGRP and CGRP receptor distribute differently in TG neurons and nerve fibers [10,60], we then detected SFKs distribution pattern in mouse TG and whether SFKs co-localize with CGRP or RAMP1. One C57BL6/J mouse was anesthetized in depth in 5% isoflurane with O_2_:N_2_O (1:2) and transcardially perfused with phosphate buffer saline (09–8912-100, Medicago, Uppsala, Sweden) and 4% paraformaldehyde (P804537, Macklin, Shanghai, China). The TG were collected and post-fixed in 4% paraformaldehyde at 4 °C overnight. The tissues were dehydrated in 10%, 20%, and 30% sucrose (V900116, Sigma-Aldrich, St. Louis, MO, USA) solutions at 4 °C overnight. Before sectioning, the tissues were embedded in Tissue-Tek O.C.T. Compound (4583, Sakura, Flemingweg, The Netherlands). Coronal sections at 20 μm of the TG tissues were prepared using a cryostat (CM1950, Leica, Tokyo, Japan) and fixed on glass slides. The TG slice was permeabilized in 0.25% Triton X-100 (V90050210, Sigma-Aldrich, St. Louis, MO, USA) for 15 min and blocking in 2% donkey serum (D9663, Sigma-Aldrich) with 2% bovine serum albumin (V900933, Sigma-Aldrich) and 0.1% Tween20 (P1379, Sigma-Aldrich) for 1.5 h at room temperature. Subsequently, the TG slice was incubated with anti-SFKs antibody (1:40, AF3389, R&D Systems, Minneapolis, MN, USA) with anti-CGRP antibody (1:50, ab81887, Abcam, Cambridge, UK) or anti-RAMP1 antibody (1:50, ARR-021, Alomone Labs, Jerusalem, Israel) respectively at 4 °C overnight. The next day, the TG slice was incubated with Alexa fluor 488 donkey anti-goat secondary antibody (1:500, A11055, Invitrogen, Carlsbad, CA, USA) with 568 goat anti-mouse secondary antibody (1:500, A11004, Invitrogen) or 568 donkey anti-rabbit secondary antibody (1:500, A10042, Invitrogen) at room temperature for 1 h in the dark, after which they were incubated in 4′,6-diamidino-2-phenylindole (DAPI, 1:5000, D8417, Sigma-Aldrich) for 5 min. After mounting in mounting solution (S36936, Invitrogen), the expressions of SFKs, CGRP, and RAMP1 in the TG slice were imaged using a Confocal Laser Scanning Microscope (LSM880, Zeiss, Jena, Germany). The co-localization of SFKs and CGRP or RAMP1 in the acquired images was analyzed qualitatively.

### 2.8. Statistical Analysis

For quantitative studies, all raw data generated in experiments were statistically analyzed using GraphPad Prism 7.0 (San Diego, CA, USA) for testing if each dataset followed normal distribution and if significant difference existed between the data of two comparable experimental groups. In order to choose a proper test for analyzing significant statistical difference between two groups, a Shapiro–Wilk test was performed for all the datasets to determine if they followed normal distribution. If the normality test was passed, the data were presented as mean ± standard error of the mean, and the significance of intergroup statistical difference was analyzed by two-tailed unpaired t-test; if not, the data were presented as median (interquartile range), and the significance of intergroup statistical difference was analyzed by two-tailed Mann–Whitney test. Four types of significant intergroup statistical difference were used: * *p* < 0.05, ** *p* < 0.01, *** *p* < 0.001, or **** *p* < 0.0001. Detailed data presentation and statistical analysis for each quantitative study was described in the respective figure legend.

## 3. Results

### 3.1. pYEEI Alone Did Not Increase CGRP Release and IL-1β Gene Expression in the Mouse TG

**Figure 1:**
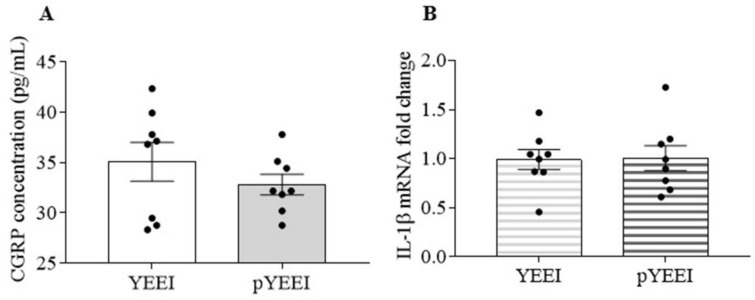
pYEEI alone did not alter CGRP release and IL-1β gene expression in the mouse TG. (**A**,**B**) Effects of 1 mM pYEEI or 1 mM YEEI (*n* = 8 per group) on CGRP release (pg/mL) and IL-1β mRNA level at 20 min post treatment. IL-1β mRNA level was present in the fold change relative to the geometric mean of β-actin and PPIA mRNA levels. Two-tailed unpaired *t*-test was used for the comparison in CGRP release and IL-1β mRNA level between the YEEI group and the pYEEI group.

We examined whether activation of SFKs increases CGRP release and IL-1β gene expression in the TG. When treated the TG with 1 mM pYEEI, the SFKs activator, the CGRP level was 32.8 ± 1 pg/mL, which was not significantly different from the CGRP level at 35.1 ± 1.9 pg/mL in the YEEI group (*n* = 8 per group, Figure 1A). Similarly, 1 mM pYEEI did not affect the IL-1β mRNA level either, which was 1 ± 0.1 (vs. 1 ± 0.1 in the YEEI group, Figure 1B) (*n* = 8 per group).

### 3.2. Saracatinib Reduced CGRP Release Induced by H_2_O_2_ in the Mouse TG

**Figure 2:**
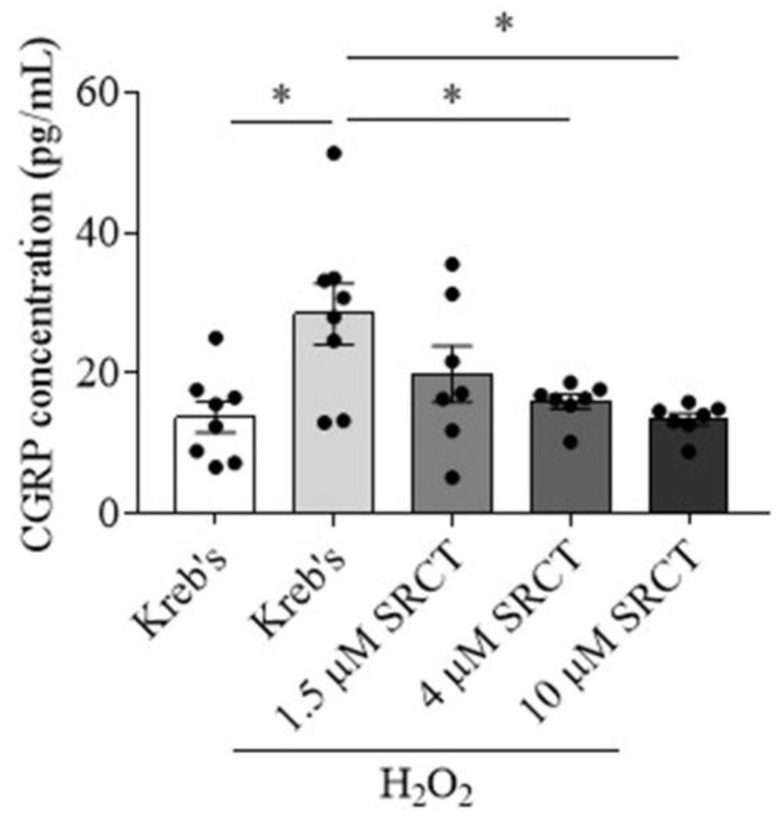
Saracatinib reduced CGRP release induced by H_2_O_2_ in the mouse TG. Effects of Kreb’s (*n* = 8); 1 mM H_2_O_2_ (*n* = 8); 1.5 μM (*n* = 7), 4 μM (*n* = 7), or 10 μM (*n* = 7) saracatinib in the presence of 1 mM H_2_O_2_ at 20 min post treatment on CGRP release (pg/mL). Abbreviations: saracatinib (SRCT). Two-tailed unpaired *t*-test was used for the comparison in CGRP release between the H_2_O_2_ group and either the Kreb’s group or the saracatinib in the presence of H_2_O_2_ group. Significant differences were labeled as * *p* < 0.05.

This section determined whether deactivation of SFKs reduces CGRP release induced by ROS in the TG. H_2_O_2_ at 1 mM increased the level of CGRP in the TG culture medium to 28.4 ± 4.4 pg/mL in comparison with that at 13.7 ± 2.2 pg/mL in the Kreb’s group (*n* = 8 per group, *p* = 0.0126, Figure 2). In the presence of 1 mM H_2_O_2_, 1.5 µM saracatinib (*n* = 7), the SFKs inhibitor, slightly reduced the CGRP level from the TG to 19.8 ± 4 pg/mL, which was not significantly different from that in the H_2_O_2_ group (Figure 2). When saracatinib was applied at 4 µM (*n* = 7), a significant reduction in the CGRP level to 15.9 ± 1 pg/mL was seen compared to that in the H_2_O_2_ group (*p* = 0.024, Figure 2). Saracatinib at 10 µM (*n* = 7) also significantly reduced the level of CGRP to 13.4 ± 0.9 compared to that in the H_2_O_2_ group (*p* = 0.0105, Figure 2). These data supported a concentration-response effect of saracatinib on CGRP release from the TG primed by H_2_O_2_.

### 3.3. Saracatinib Reduced IL-1β, CCL2, and CXCL1 Release Induced by CGRP in the Mouse TG

**Figure 3:**
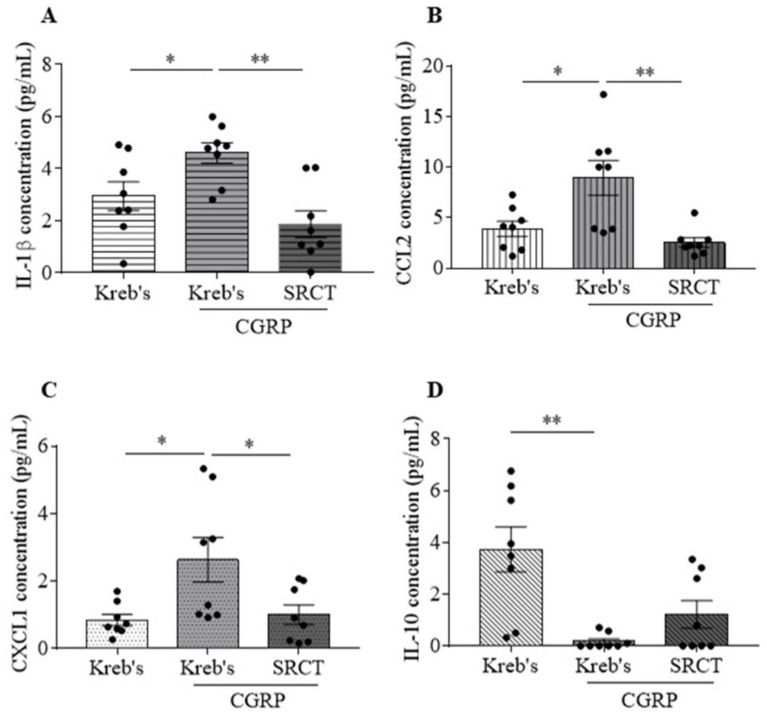
Saracatinib reduced IL-1β, CCL2, and CXCL1 release induced by CGRP in the mouse TG. (**A**–**D**) Effects of Kreb’s, 3 µM CGRP, or 1.5 μM saracatinib in the presence of 3 μM CGRP (*n* = 8 per group) at 20 min post treatment on IL-1β, CCL2, CXCL1, and IL-10 release (pg/mL). Abbreviations: saracatinib (SRCT). Two-tailed unpaired *t*-test was used for the comparison in IL-1β, CCL2, CXCL1, and IL-10 release between the CGRP group and either the Kreb’s group or the saracatinib in the presence of CGRP group. Significant differences were labeled as * *p* < 0.05 or ** *p* < 0.01.

We addressed whether deactivation of SFKs reduces inflammatory cytokine release induced by CGRP in the TG. Among the 12 pro-inflammatory cytokines (CCL2, CCL5, CXCL1, CXCL10, GM-CSF, IFN-α, IFN-β, IFN-γ, IL-1β, IL-6, IL-12, TNFα) and one anti-inflammatory cytokine (IL-10), 3 µM CGRP promoted the levels of IL-1β to 4.6 ± 0.4 pg/mL (vs. 2.9 ± 0.5 pg/mL in the Kreb’s group, *p* = 0.0288), CCL2 to 8.9 ± 1.7 pg/mL (vs. 3.9 ± 0.7 pg/mL in the Kreb’s group, *p* = 0.0232), and CXCL1 to 2.6 ± 0.7 pg/mL (vs. 0.9 ± 0.2 pg/mL in the Kreb’s group, *p* = 0.0303) in the TG culture medium (*n* = 8 per group, Figure 3A–C). Differently, 3 µM CGRP decreased the level of IL-10 to 0.2 ± 0.1 pg/mL (vs. 3.7 ± 0.9 pg/mL in the Kreb’s group, *p* = 0.0017, *n* = 8 per group, Figure 3D) in the medium. It is noted that 3 µM CGRP did not alter the levels of the other 9 cytokines (Supplementary Figure S1). As expected, in the presence of 3 µM CGRP, 1.5 µM saracatinib decreased the levels of IL-1β to 1.9 ± 0.5 pg/mL (*p* = 0.001), CCL2 to 2.5 ± 0.5 pg/mL (*p* = 0.0069), and CXCL1 to 1 ± 0.3 pg/mL (*p* = 0.0476) when compared to the respective data in the CGRP group (*n* = 8 per group, Figure 3A–C). However, 1.5 µM saracatinib did not significantly affect the reduced level of IL-10 elicited by 3 µM CGRP, which was 1.2 ± 0.5 (*n* = 8 per group, Figure 3D).

### 3.4. Saracatinib Reduced IL-1β and CCL2 Gene Expression Induced by CGRP in the Mouse TG

**Figure 4:**
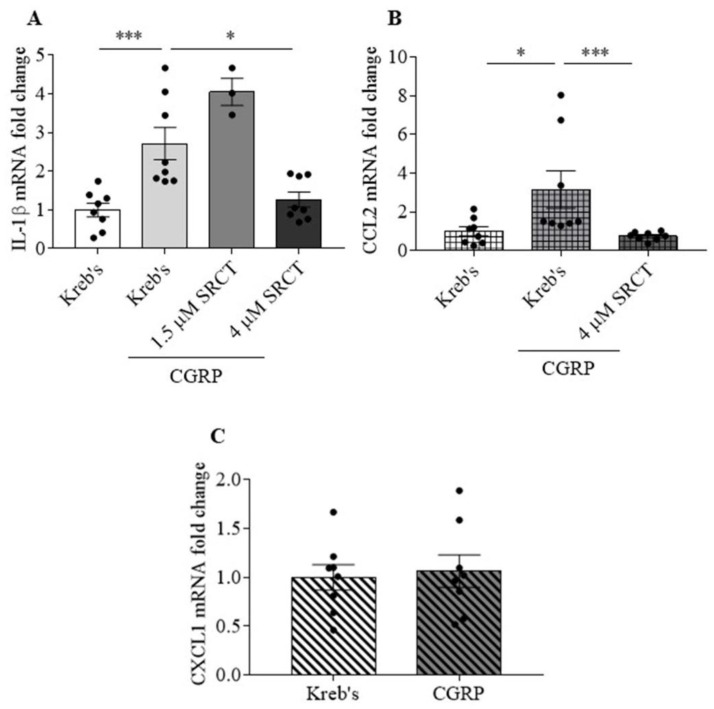
Saracatinib reduced IL-1β, CCL2 and CCL1 gene expression induced by CGRP in the mouse TG. (**A**–**C**) Effects of Kreb’s, 3 µM CGRP, 1.5 μM, or 4 μM saracatinib in the presence of 3 μM CGRP (*n* = 8 per group) at 60 min post treatment on mRNA level of IL-1β, CCL2 and CCL1 in respective order. IL-1β, CCL2, and CCL1 mRNA levels were present in the fold change relative to the geometric mean of β-actin and PPIA mRNA levels. Abbreviations: saracatinib (SRCT). Two-tailed unpaired Mann–Whitney test was used for the comparison in IL-1β and CCL2 mRNA levels between the CGRP group and either the Kreb’s group or the saracatinib in the presence of CGRP group. Significant differences were labeled as * *p* < 0.05 or *** *p* < 0.001.

Our data demonstrated that saracatinib reduced IL-1β, CCL2, and CXCL1 release promoted by CGRP in the TG (Figure 3A–C). All these proteins are associated with pain hypersensitivity and migraine [61,62,63,64,65]. We therefore further investigated whether SFKs activity promotes cytokines production machinery by examining the effect of the SFKs inhibitor saracatinib on their CGRP-induced gene expression. CGRP at 3 µM increased the mRNA levels of IL-1β to 2.1 (2.1) (vs. 1.1 (0.9) in the Kreb’s group, *p* = 0.0003) and CCL2 to 1.5 (4.5) (vs. 0.9 (1.2) in the Kreb’s group, *p* = 0.0207) in the TG (*n* = 8 per group, Figure 4A,B). In contrast, 3 µM CGRP did not affect the CXCL1 mRNA level, which was 1.1 ± 0.2 compared to that at 1 ± 0.1 in the Kreb’s group (*n* = 8 per group, Figure 4C). When 1.5 µM saracatinib was applied in the presence of 3 µM CGRP, the IL-1β mRNA level was 4 (1.2), which was insignificantly different from that in the CGRP group (Figure 4A). Saracatinib at 4 µM, however, resulted in a pronounced reduction in the mRNA levels of IL-1β to 1 (1.1) (*p* = 0.0148) and CCL2 to 0.8 (0.3) (*p* = 0.0002) in comparison with that in the CGRP group (*n* = 8 per group, Figure 4A,B).

### 3.5. The Protein Level of Phosphorylated SFKs at Y416 Was Increased by H_2_O_2_, Which Was Reduced by BIBN4096 in the Mouse TG

**Figure 5:**
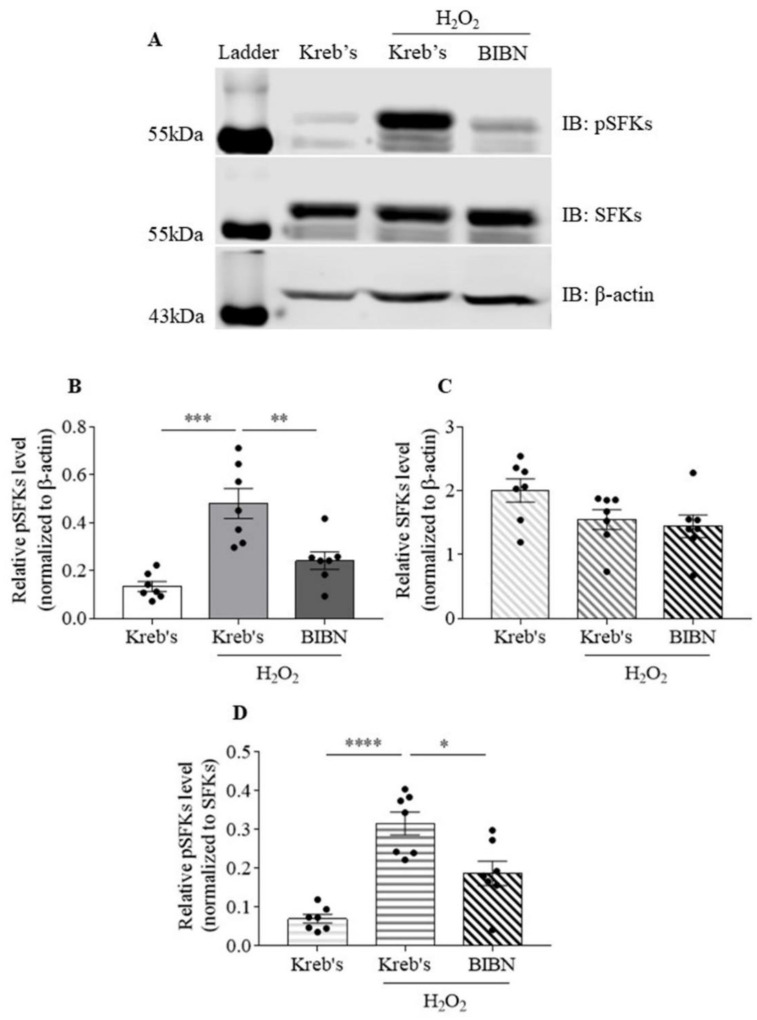
The protein level of phosphorylated SFKs at Y416 was increased by H_2_O_2_ in the mouse TG. (**A**) The representative Western blot bands of phosphorylated SFKs at Y416, SFKs, and β-actin subjected to the treatment with Kreb’s, 1 mM H_2_O_2_, or 10 µM BIBN4096 in the presence of 1 mM H_2_O_2_. (**B**–**D**) Effects of Kreb’s, 1 mM H_2_O_2_, or 10 µM BIBN4096 in the presence of 1 mM H_2_O_2_ (*n* = 7 per group) at 20 min post treatment on the protein levels of phosphorylated SFKs at Y416 and SFKs relative to that of β-actin and on the protein level of phosphorylated SFKs at Y416 relative to that of SFKs, all of which were presented in the absolute ratio. Abbreviations: BIBN4086 (BIBN), phosphorylated SFKs at Y416 (pSFKs). Two-tailed unpaired *t*-test was used for the comparison in the protein level of phosphorylated SFKs at Y416 between the H_2_O_2_ group and either the Kreb’s group or the BIBN4096 in the presence of 1 mM H_2_O_2_ group. Significant differences were labeled as * *p* < 0.05, ** *p* < 0.01, *** *p* < 0.001, or **** *p* < 0.0001. Original western blot images for the representative images in Figure 5 was shown in Supplementary Figure S2.

We examined whether SFKs activity is increased by H_2_O_2_ and whether such elevation can be reversed by inhibition of CGRP receptor using BIBN4096. When the protein level of phosphorylated SFKs at Y416 was normalized to that of β-actin, exposure to 1 mM H_2_O_2_ increased the protein level of phosphorylated SFKs at Y416 to 0.48 ± 0.06 compared to that at 0.13 ± 0.02 in the Kreb’s group (*n* = 7 per group, *p* = 0.0010, Figure 5B). BIBN4096 at 10 µM reduced the H_2_O_2_-enhanced protein level of phosphorylated SFKs at Y416 to 0.24 ± 0.04 compared to that in the H_2_O_2_ group (*n* = 7 per group, *p* = 0.0082, Figure 5B). In contrast, the protein level of SFKs was unchanged among the three groups (Figure 5C), suggesting that the protein level of SFKs was insensitive to H_2_O_2_ or BIBN4096. When the protein level of phosphorylated SFKs at Y416 was normalized to that of SFKs, consistently, the protein level of phosphorylated SFKs at Y416 was increased to 0.32 ± 0.03 by H_2_O_2_ in comparison with that at 0.07 ± 0.01 in the Kreb’s group (*n* = 7 per group, *p* < 0.0001, Figure 5D). BIBN4096 reduced H_2_O_2_-enhanced protein level of phosphorylated SFKs at Y416 to 0.19 ± 0.03 in comparison with that in the H_2_O_2_ group (*n* = 7 per group, *p* = 0.0117, Figure 5D).

### 3.6. The Protein Level of Phosphorylated SFKs at Y416 Was Increased by CGRP, Which Was Reduced by Both PKI (14-22) Amide and BIBN4096 in the Mouse TG

**Figure 6:**
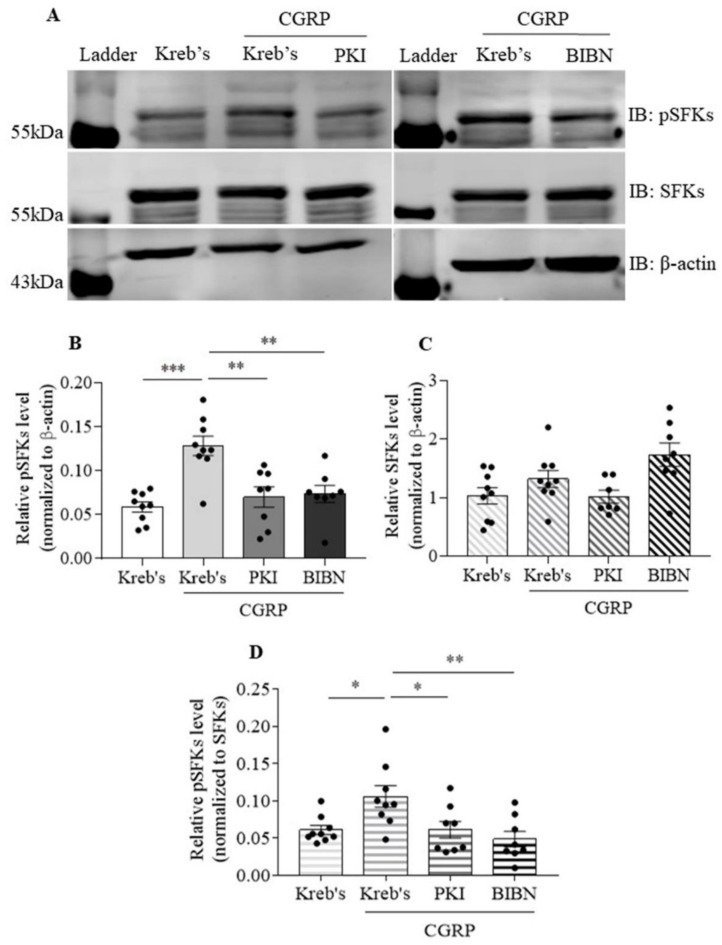
The protein level of phosphorylated SFKs at Y416 was increased by CGRP, which was reduced by both PKI (14-22) Amide and BIBN4096 in the mouse TG. (**A**) The representative Western blot bands of phosphorylated SFKs at Y416, SFKs, and β-actin subjected to the treatment with Kreb’s, 3 µM CGRP, 30 µM PKI (14-22) Amide or 10 µM BIBN4096 in the presence of 3 µM CGRP for 20 min. (**B**–**D**) Effects of Kreb’s (*n* = 9), 3 μM CGRP (*n* = 9), 30 μM PKI (14-22) Amide (*n* = 8) or 3 µM BIBN4096 (*n* = 8) in the presence of 3 μM CGRP on the protein levels of phosphorylated SFKs at Y416 and SFKs relative to that of β-actin and on the protein level of phosphorylated SFKs at Y416 relative to that of SFKs, all of which were presented in the absolute ratio. Abbreviations: PKI (14-22) Amide (PKI), BIBN4086 (BIBN), phosphorylated SFKs at Y416 (pSFKs). Two-tailed unpaired *t*-test was used for the comparison in the protein level of phosphorylated SFKs at Y416 between the CGRP group and either the Kreb’s group, the PKI (14-22) Amide, or the BIBN4096 in the presence of CGRP groups. Significant differences were labeled as * *p* < 0.05, ** *p* < 0.01, or *** *p* < 0.001. Original western blot images for the representative images in Figure 6 were shown in Supplementary Figures S3 and S4.

We next investigated whether SFKs activity can be elevated by CGRP and whether such elevation is sensitive to PKA or CGRP receptor inhibition using PKI (14-22) Amide and BIBN4096, respectively. When the protein level of phosphorylated SFKs at Y416 was normalized to that of β-actin, 3 µM CGRP markedly increased the protein level of phosphorylated SFKs at Y416 to 0.13 ± 0.01 compared to that at 0.06 ± 0.01 in the Kreb’s group (*n* = 9 per group, *p* = 0.0001, Figure 6B). In the presence of 3 µM CGRP, both 30 µM PKI (14-22) Amide (*n* = 8) and 3 µM BIBN4096 (*n* = 8) reduced the protein level of phosphorylated SFKs at Y416 to 0.07 ± 0.01 compared to that in the CGRP group (*p* = 0.0023 and 0.002 respectively, Figure 6B). In contrast, the protein level of SFKs was unchanged among the four groups (Figure 6C). Consistently, when the protein level of phosphorylated SFKs at Y416 was normalized to that of SFKs, the protein level of phosphorylated SFKs at Y416 was increased to 0.11 ± 0.01 by CGRP in comparison with that at 0.06 ± 0.01 in the Kreb’s group (*n* = 9 per group, *p* = 0.0158, Figure 6D). In the presence of CGRP, both PKI (14-22) Amide (*n* = 8) and BIBN4096 (*n* = 8) reduced the protein level of phosphorylated SFKs at Y416 to 0.06 ± 0.01 and 0.05 ± 0.01 in comparison with that in the CGRP alone group (*p* = 0.0283 and 0.0063, respectively, Figure 6D).

### 3.7. The Protein Levels of Phosphorylated SFKs at Y416 and Released Cytokines Induced by CGRP Were Positively Correlated in the Mouse TG

**Figure 7:**
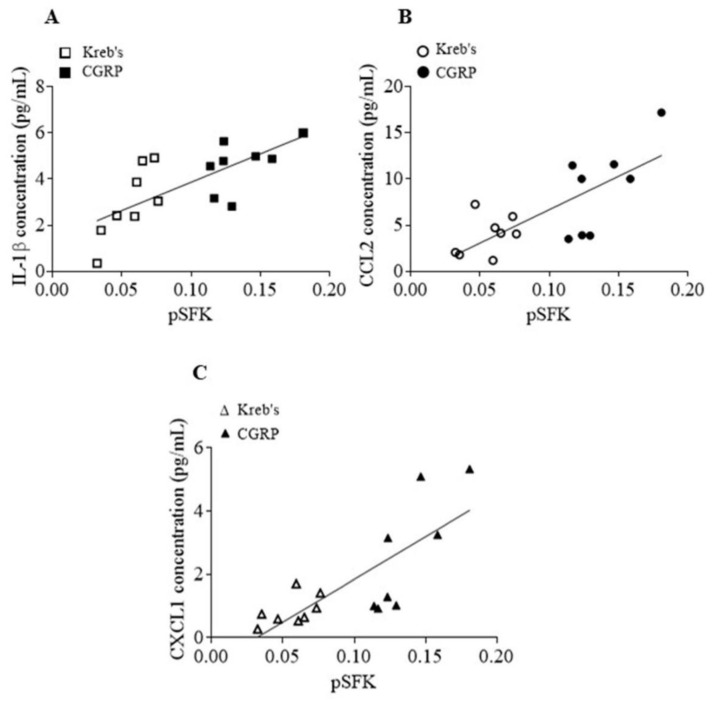
The levels of phosphorylated SFKs at Y416 and released cytokines induced by CGRP were correlated in the mouse TG. Correlation analysis between the levels of phosphorylated SFKs at Y416 and IL-1β (**A**), CCL2 (**B**), CXCL1 (**C**) release in the mouse TG treated by Kreb’s and 3 µM CGRP (*n* = 8 per group) for 20 minutes.

We then carried out further analysis to explore whether the SFKs activity and cytokine release enhanced by CGRP are correlated. A positive relationship between the increased levels of phosphorylated SFKs at Y416 and IL-1β release (*r* = 0.7261, *p* = 0.0014, Figure 7A), CCL2 release (*r* = 0.7462, *p* = 0.0009, Figure 7B), and CXCL1 release (*r* = 0.7768, *p* = 0.0004, Figure 7C) was seen in the TG treated by both Kreb’s and 3 µM CGRP (*n* = 8 per group).

### 3.8. SFKs Co-Localized with CGRP and RAMP1 in the Mouse TG

**Figure 8:**
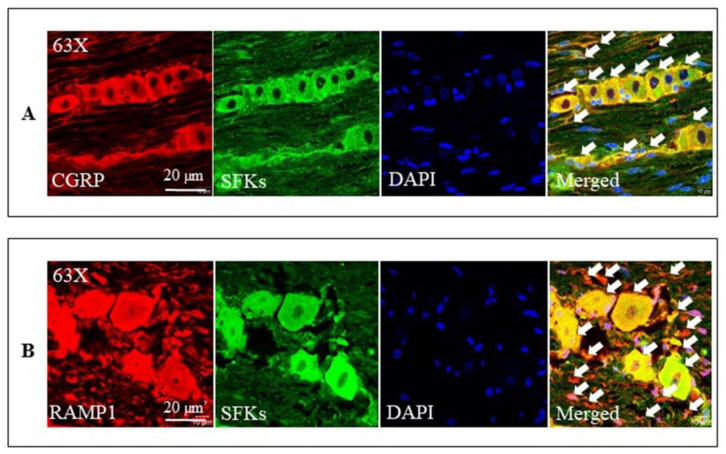
SFKs co-localized with both CGRP and RAMP1 in the mouse TG. (**A**) The representative images of double staining with the anti-CGRP antibody and anti-SFKs antibody in the TG. (**B**) The representative images of double staining with the anti-RAMP1 antibody and anti-SFKs antibody in the TG. CGRP and RAMP1 were stained with the anti-CGRP antibody and anti-RAMP1 antibody, respectively, and are shown in red; SFKs were stained with the anti-SFKs antibody and are shown in green; nucleus was stained with DAPI and is shown in blue. Co-localization of SFKs and CGRP or RAMP1 is shown in yellow and is indicated by the white arrows.

Consistent with the previous reports [10,66,67,68], we were able to demonstrate that CGRP immunoreactivity was present in small to medium-sized neurons and C fibers (Figure 8A) while RAMP1 immunoreactivity was present in large neurons and Aδ fibers (Figure 8B) in the TG. Further, SFKs immunoreactivity was present in the neurons of nearly all sizes and fibers in the TG (Figure 8A,B). Double staining with the anti-SFKs antibody and anti-CGRP antibody or anti-RAMP1 antibody demonstrated that SFKs co-localized with both CGRP and RAMP1 proteins in their respective cell types and fibers of the TG (Figure 8A,B).

## 4. Discussion

Our data demonstrate that SFKs activity facilitates the crosstalk between CGRP and cytokines in sensitizing trigeminal ganglion by transmitting CGRP receptor/PKA signaling. These findings uncover an unprecedented role of SFKs in migraine pain transmission.

We have previously demonstrated that SFKs mediate CGRP release and IL-1β gene expression and SFKs inhibition by saracatinib reduces the stress-sensing cation channel transient receptor potential ankyrin 1 (TRPA1)-activated CGRP release and IL-1β gene expression in the mouse TG [21]. We therefore postulated that direct SFKs activation may induce CGRP release and neuroinflammation from the TG thus triggering TG activation. Unexpectedly in this communication, activation of SFKs using pYEEI does not alter CGRP release or IL-1β gene expression in the mouse TG (Figure 1). pYEEI, the SFKs activator, activates SFKs by binding to their SH2 domains, which disrupts the closing conformation of inactive SFKs ensuing their open active conformation [34,37,38]. Given that the concentration (1 mM) of pYEEI applied in this study is high enough to trigger neuronal activation [35,36], the current data suggest that SFKs activation by pYEEI alone is least likely to be a stimulus for TG activation, which is unlike other stimuli such as KCl or TRPA1 activator that can activate multiple pathways triggering mass activation and sensitization of TG [21].

We then explored whether modulating SFKs activity may be only effective when the TG is pre-primed. Indeed, in the mouse TG that is pre-sensitized by H_2_O_2_, the SFKs inhibitor, saracatinib, markedly reduces CGRP release from TG in a concentration-dependent manner (Figure 2). Consistent with this, SFKs activity is increased by H_2_O_2_ in the mouse TG (Figure 5). These data highlight a key role of SFKs activity in facilitating TG sensitization by mediating endogenous CGRP release. Moreover, these data extend the previous findings that deactivation of SFKs reverses CGRP release promoted by nerve growth factor and capsaicin in dorsal root ganglion neurons [20] and by a TRPA1 activator, umbellulone, in the TG [21]. 

Similar to the release of CGRP, the SFKs inhibitor saracatinib also reduces the release of IL-1β, CCL2, and CXCL1 from the mouse TG pre-sensitized by exogenous CGRP (Figure 3), which consistently increases SFKs activity (Figure 6). The levels of all these three cytokines released show positive correlations with the level of respective phosphorylated SFKs induced by CGRP (Figure 7), highlighting the importance of SFK activity in promoting TG neuroinflammation. Moreover, these data extend the previous finding that SFKs activity contributes to the release of inflammatory cytokines in astrocytes [25] and microglia [22,23,24]. Among these inflammatory cytokines, IL-1β potentiates the excitability of nociceptive neurons in the TG and directly causes the hypersensitivity to nociception ensuing the nociceptive behaviors, hyperalgesia, and allodynia [69]. While IL-1β has a well-identified role in migraine pathogenesis [65], CXCL1 [61,62,70,71] and CCL2 [63,64,72] are associated with pain hypersensitivity and more recently with migraine. It is therefore concluded that SFKs activity can synergistically elevate CGRP and cytokine release to reinforce TG sensitization and facilitate pain transmission. It is noted that, unlike the three inflammatory cytokines, the anti-inflammatory cytokine, IL-10, release is insensitive to SFK inhibition. This might suggest that SFKs activity plays a more prominent role in controlling inflammatory cytokine but not anti-inflammatory cytokine release.

One question to ask from our data is how the SFKs-mediated TG sensitization is sustained. In our study, besides cytokine release by CGRP being reduced by saracatinib at 20 min post treatment (Figure 3), the induction of IL-1β and CCL2 gene expression by exogenous CGRP was also reduced by SFKs deactivation at 1 h post treatment in the mouse TG (Figure 4). These data are consistent with our previous finding that SFKs activity contributes to IL-1β gene expression induced by the TRPA1 activator umbellulone in the mouse TG [21]. It is highly likely that the SFKs-induced transcriptional machinery activation of these cytokines is crucial to sustain the TG sensitization, especially in that IL-1β mRNA expression and protein expression are well correlated [73]. The possible mechanism by which SFKs mediate cytokines gene expression could be via transcription factors or histone modification. SFKs are known to activate the transcription factor NFκB in models of neurodegenerative diseases [25,74], which is important for inflammatory responses [75]. Interestingly, SFKs-mediated CCL2 gene expression and histone H3 acetylation at the CCL2 promoter are associated in macrophages [76]. Furthermore, CGRP is a potent neuroinflammatory mediator that induces the expression and release of cytokines in the TG [11,12,13,14], including IL-1β, CCL2, CXCL1, all of which in turn stimulate CGRP release [16,77], thereby inducing a positive feedback loop of TG sensitization. Similar to CGRP, IL-1β can activate SFKs in several cell lines [78,79,80,81], which suggests that released cytokines are highly likely to strengthen SFKs activity again to induce CGRP release and aggravate TG sensitization. This is consistent with the significant positive correlation between the elevated SFKs activity and cytokine release induced by CGRP, which supports the model that SFKs activity facilitates the crosstalk between CGRP and cytokines (Figure 7). Taken together, we propose a feedback mechanism by which SFKs activity facilitates the crosstalk and intraganglionic signaling between CGRP and cytokines in stress-primed TG to potentiate TG sensitization and ultimately trigeminovascular sensitization. 

**Figure 9:**
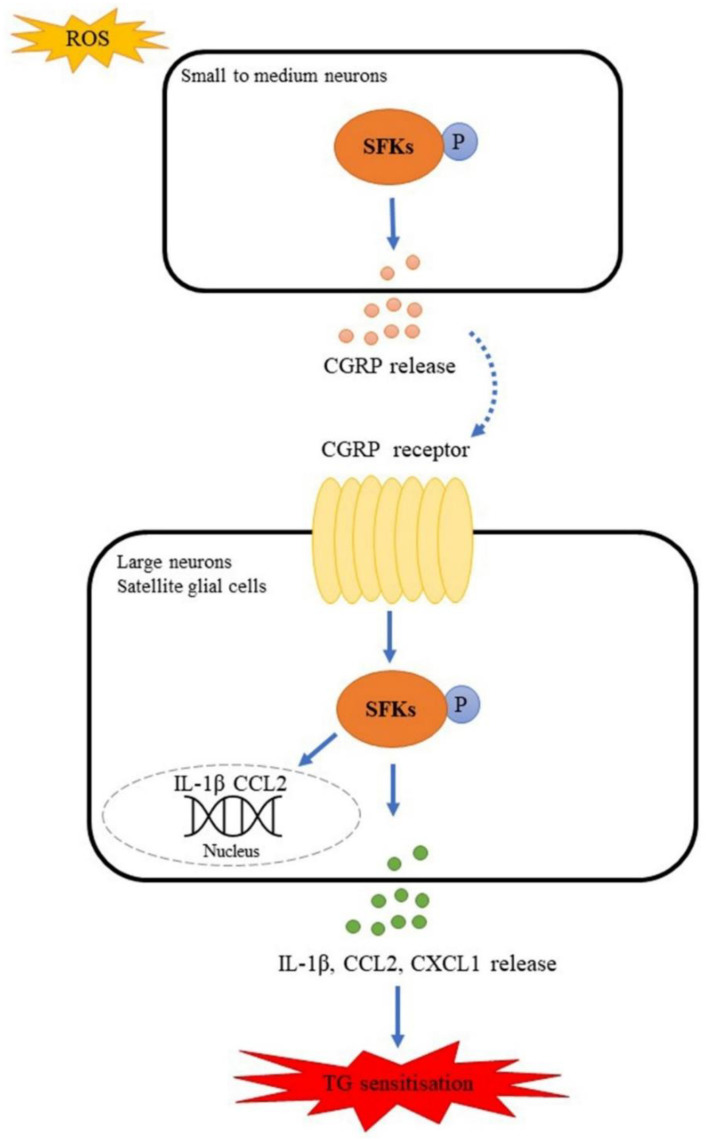
Model of SFKs activity facilitating the crosstalk between CGRP and cytokines by transmitting CGRP receptor signaling to potentiate TG sensitization. SFKs are activated in response to ROS to induce CGRP release in small to medium neurons; released CGRP binds to CGRP receptor (dotted line with arrow) to activate SFKs in large neurons and satellite glial cells, which causes IL-1β, CCL2, CXCL1 release and IL-1β, CCL2 gene expression, thus leading to TG sensitization.

The molecular mechanism underlying the SFKs-mediated crosstalk between CGRP and cytokines in sensitizing TG has yet to be fully defined. Interestingly, SFKs activation induced by either H_2_O_2_ (Figure 5) or exogenous CGRP (Figure 6) can be reduced by the CGRP receptor inhibitor, BIBN4096, in the mouse TG, supporting CGRP receptor-dependent SFKs activation in TG. As SFKs co-localize with CGRP in small- to medium-sized neurons and C fibers, whilst RAMP1 in large neurons, Aδ fibers, and satellite glial cells of the mouse TG (Figure 8), we can conclude that the SFKs-mediated crosstalk between CGRP and cytokines is dependent on CGRP/CGRP receptor signaling. Notably, PKA is known to actively transmit CGRP/CGRP receptor signaling to initiate downstream signaling cascades, thus leading to TG activation [52,53,54]. PKA robustly increases SFKs activity in cell lines [55], spinal dorsal horn [56], and hypothalamic arcuate nucleus neurons [36], and PKA/SFKs pathway facilitates neuronal firing [56] and pain sensitivity [36]. This can be compared with our previous study which demonstrates that SFKs activity is elevated by PKA upon TRPA1 activation to promote CGRP release in the mouse TG [21]. Furthermore, in the present study, the enhanced phosphorylated SFKs at Y416 induced by CGRP is also reduced by the PKA inhibitor PKI (14-22) Amide, which is similar to that by the CGRP receptor inhibitor BIBN4096 (Figure 6). Taken together, these data pinpoint that SFKs activity is increased downstream of CGRP/CGRP receptor signaling via PKA activity in the TG, thereby contributing to CGRP-cytokines crosstalk and TG sensitization (Figure 9). Given that PKA promotes the phosphorylation of SFKs at the S17 site followed by autophosphorylation at their Y416 site [55], it is likely that SFKs are activated directly by PKA downstream of CGRP/CGRP receptor signaling in the TG, which awaits future validation. Future work should also examine whether CGRP co-localizes with PKA in the TG.

In this study, only male mice are used to explore the role of SFKs in TG sensitization so that the effect of hormonal fluctuation in females can be minimized. Similarly, the previous studies on investigating the role of SFKs in the CSD-induced migraine with aura model [82] and the inflammatory soup-induced chronic migraine model [26] only use male rodents. Interestingly, SFKs deactivation does not show sex difference in brain perfusion abnormalities in the genetic migraine with aura model FHM2 [27]. Nevertheless, future work should explore whether SFKs mediate different migraine pathogenesis in females in order to understand if there are gender-specific effects of targeting SFKs.

## 5. Conclusions

The present study demonstrates that SFKs activity plays a pivotal role in facilitating the crosstalk between CGRP and cytokines by transmitting CGRP receptor/PKA signaling to potentiate TG sensitization and ultimately trigeminovascular sensitization. These findings shed light on the SFKs-mediated peripheral mechanism of migraine pathogenesis and support the promising efficacy of drugs targeting SFKs for migraine therapy.

## Data Availability

Not applicable.

## Supplementary Materials

The following supporting information can be downloaded at: https://www.mdpi.com/article/10.3390/cells11213498/s1. Figure S1: CGRP did not alter the release of CCL5, CXCL10, GM-CSF, IFN-α, IFN-β, IFN-γ, IL-6, IL-12, or TNFα in the mouse TG; Figure S2: Original western blot images for the representative images presented in Figure 5; Figures S3 and S4: Original western blot images for the representative images presented in Figure 6.
