# Supplementary material for: Src Family Kinases Facilitate the Crosstalk between CGRP and Cytokines in Sensitizing Trigeminal Ganglion via Transmitting CGRP Receptor/PKA Pathway
Source: Cells. 2022 Nov 4;11(21):3498. doi: 10.3390/cells11213498 (PMC9655983; doi:10.3390/cells11213498)
Supplement: Supplementary file 1 [file cells-11-03498-s001.zip › Supple Figure S1 - the rest 9 cytokines unaffected by GGRP-SFK.pptx]

## Slide 1
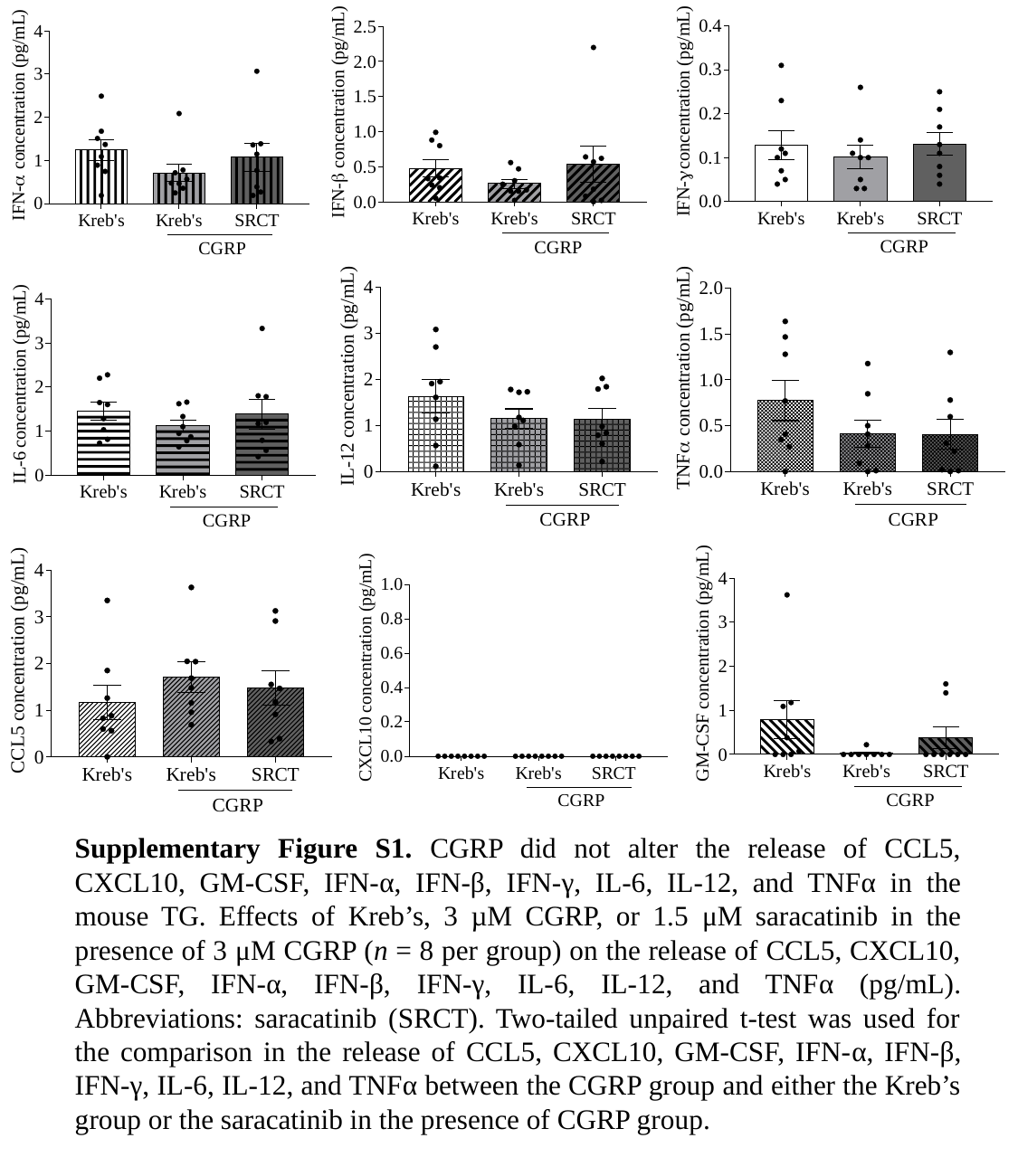

Supplementary Figure S1. CGRP did not alter the release of CCL5, CXCL10, GM-CSF, IFN-α, IFN-β, IFN-γ, IL-6, IL-12, and TNFα in the mouse TG. Effects of Kreb’s, 3 µM CGRP, or 1.5 μM saracatinib in the presence of 3 μM CGRP (n = 8 per group) on the release of CCL5, CXCL10, GM-CSF, IFN-α, IFN-β, IFN-γ, IL-6, IL-12, and TNFα (pg/mL). Abbreviations: saracatinib (SRCT). Two-tailed unpaired t-test was used for the comparison in the release of CCL5, CXCL10, GM-CSF, IFN-α, IFN-β, IFN-γ, IL-6, IL-12, and TNFα between the CGRP group and either the Kreb’s group or the saracatinib in the presence of CGRP group.
