# Supplementary material for: Src Family Kinases Facilitate the Crosstalk between CGRP and Cytokines in Sensitizing Trigeminal Ganglion via Transmitting CGRP Receptor/PKA Pathway
Source: Cells. 2022 Nov 4;11(21):3498. doi: 10.3390/cells11213498 (PMC9655983; doi:10.3390/cells11213498)
Supplement: Supplementary file 1 [file cells-11-03498-s001.zip › Supple Figure S2 - original western blots of H2O2-pSFK.pptx]

## Slide 1
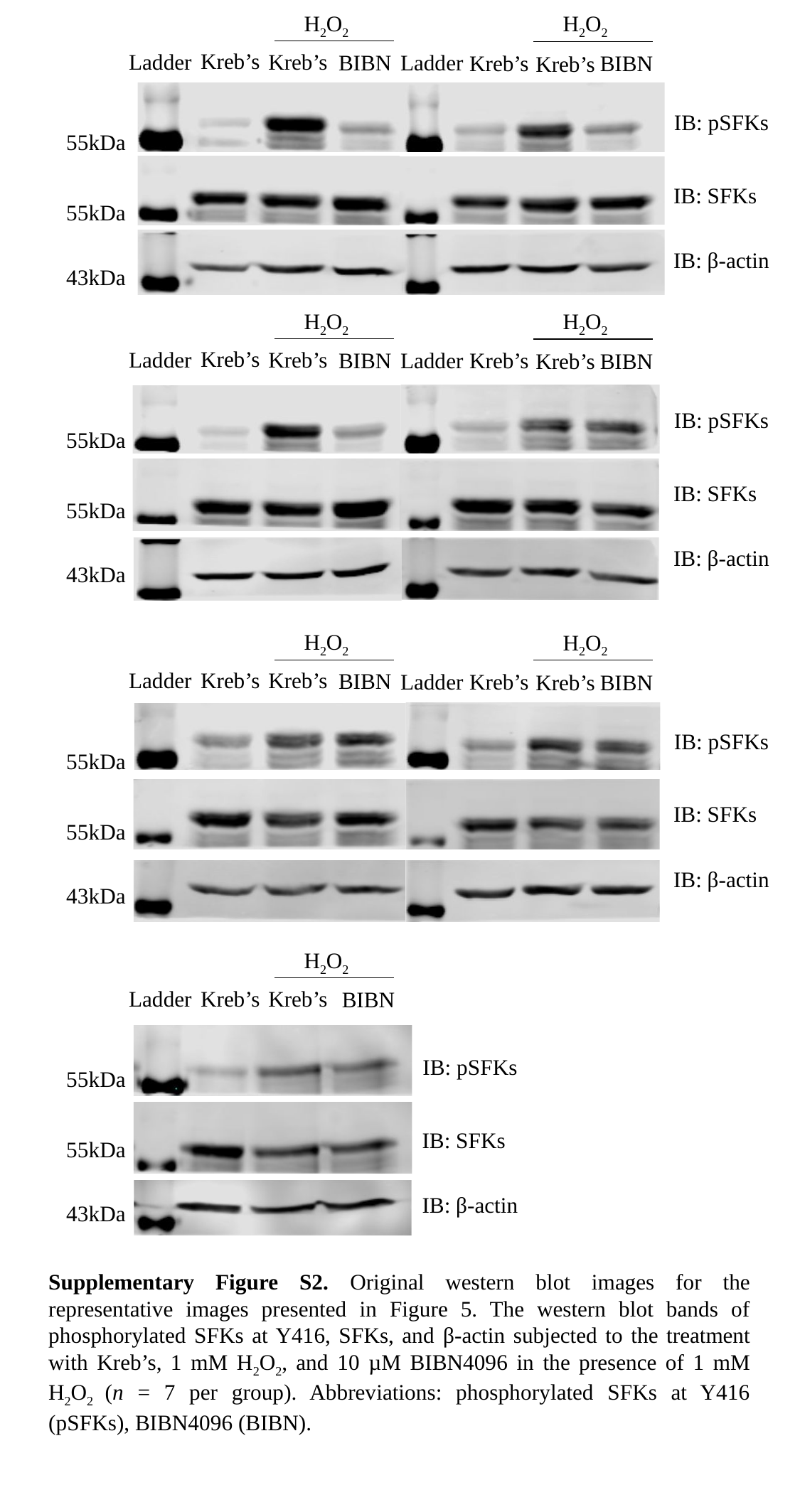

H2O2
H2O2
Kreb’s
Ladder
Kreb’s
BIBN
Ladder
Kreb’s
BIBN
Kreb’s
IB: pSFKs
55kDa
IB: SFKs
55kDa
IB: β-actin
43kDa
H2O2
H2O2
Kreb’s
Ladder
Kreb’s
BIBN
Ladder
Kreb’s
BIBN
Kreb’s
IB: pSFKs
55kDa
IB: SFKs
55kDa
IB: β-actin
43kDa
H2O2
H2O2
Kreb’s
Ladder
Kreb’s
BIBN
Ladder
Kreb’s
BIBN
Kreb’s
IB: pSFKs
55kDa
IB: SFKs
55kDa
IB: β-actin
43kDa
H2O2
Kreb’s
Ladder
Kreb’s
BIBN
IB: pSFKs
55kDa
IB: SFKs
55kDa
IB: β-actin
43kDa
Supplementary Figure S2. Original western blot images for the representative images presented in Figure 5. The western blot bands of phosphorylated SFKs at Y416, SFKs, and β-actin subjected to the treatment with Kreb’s, 1 mM H2O2, and 10 µM BIBN4096 in the presence of 1 mM H2O2 (n = 7 per group). Abbreviations: phosphorylated SFKs at Y416 (pSFKs), BIBN4096 (BIBN).
