# Supplementary material for: Src Family Kinases Facilitate the Crosstalk between CGRP and Cytokines in Sensitizing Trigeminal Ganglion via Transmitting CGRP Receptor/PKA Pathway
Source: Cells. 2022 Nov 4;11(21):3498. doi: 10.3390/cells11213498 (PMC9655983; doi:10.3390/cells11213498)
Supplement: Supplementary file 1 [file cells-11-03498-s001.zip › Supple Figure S3 - original western blots of CGRP-PKA-pSFK.pptx]

## Slide 1
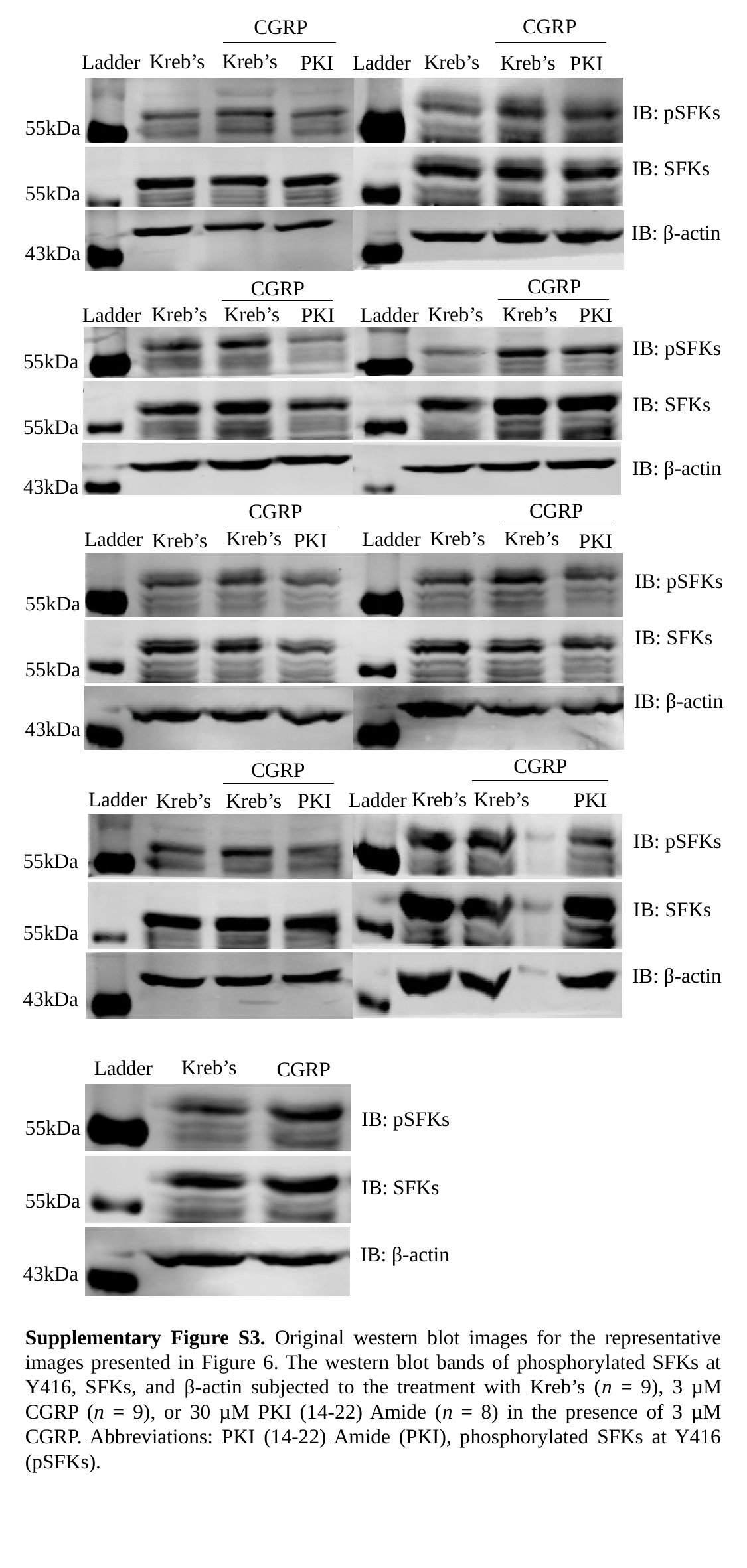

CGRP
CGRP
Kreb’s
Kreb’s
Ladder
PKI
IB: pSFKs
55kDa
IB: SFKs
55kDa
IB: β-actin
43kDa
Kreb’s
Kreb’s
Ladder
PKI
CGRP
CGRP
Kreb’s
Kreb’s
Kreb’s
Kreb’s
Ladder
Ladder
PKI
PKI
IB: pSFKs
55kDa
IB: SFKs
55kDa
IB: β-actin
43kDa
CGRP
CGRP
Kreb’s
Kreb’s
Kreb’s
Ladder
Ladder
PKI
Kreb’s
PKI
IB: pSFKs
55kDa
IB: SFKs
55kDa
IB: β-actin
43kDa
CGRP
CGRP
Kreb’s
Kreb’s
Ladder
Ladder
PKI
Kreb’s
PKI
Kreb’s
IB: pSFKs
55kDa
IB: SFKs
55kDa
IB: β-actin
43kDa
Kreb’s
Ladder
CGRP
IB: pSFKs
55kDa
IB: SFKs
55kDa
IB: β-actin
43kDa
Supplementary Figure S3. Original western blot images for the representative images presented in Figure 6. The western blot bands of phosphorylated SFKs at Y416, SFKs, and β-actin subjected to the treatment with Kreb’s (n = 9), 3 µM CGRP (n = 9), or 30 µM PKI (14-22) Amide (n = 8) in the presence of 3 µM CGRP. Abbreviations: PKI (14-22) Amide (PKI), phosphorylated SFKs at Y416 (pSFKs).
