# Supplementary material for: Src Family Kinases Facilitate the Crosstalk between CGRP and Cytokines in Sensitizing Trigeminal Ganglion via Transmitting CGRP Receptor/PKA Pathway
Source: Cells. 2022 Nov 4;11(21):3498. doi: 10.3390/cells11213498 (PMC9655983; doi:10.3390/cells11213498)
Supplement: Supplementary file 1 [file cells-11-03498-s001.zip › Supple Figure S4 - original western blots of CGRPR-pSFK.pptx]

## Slide 1
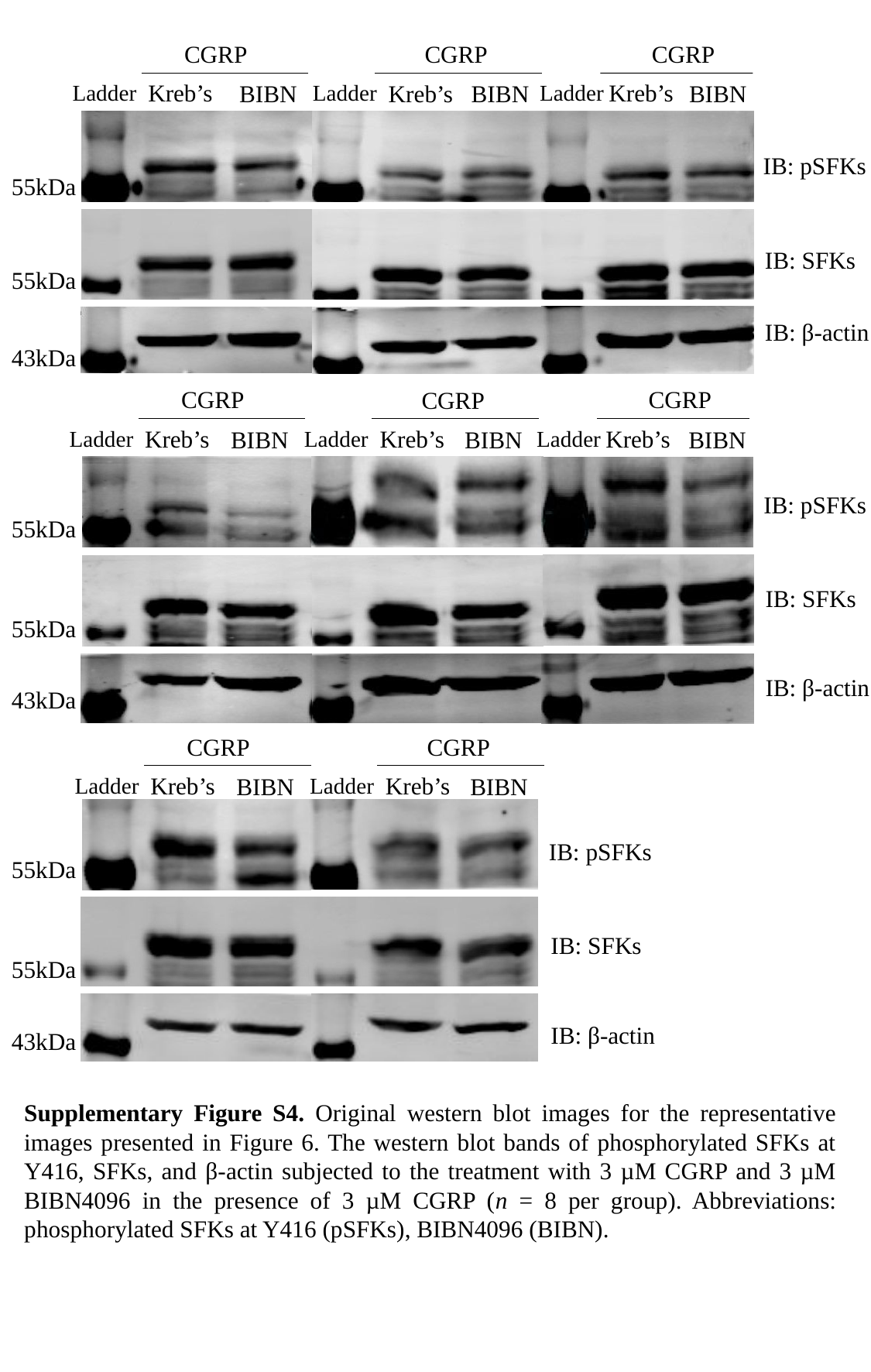

CGRP
CGRP
CGRP
Kreb’s
Kreb’s
Kreb’s
Ladder
BIBN
Ladder
BIBN
Ladder
BIBN
IB: pSFKs
55kDa
IB: SFKs
55kDa
IB: β-actin
43kDa
CGRP
CGRP
CGRP
Kreb’s
Kreb’s
Kreb’s
Ladder
BIBN
Ladder
BIBN
Ladder
BIBN
IB: pSFKs
55kDa
IB: SFKs
55kDa
IB: β-actin
43kDa
CGRP
CGRP
Kreb’s
Kreb’s
Ladder
BIBN
Ladder
BIBN
IB: pSFKs
55kDa
IB: SFKs
55kDa
IB: β-actin
43kDa
Supplementary Figure S4. Original western blot images for the representative images presented in Figure 6. The western blot bands of phosphorylated SFKs at Y416, SFKs, and β-actin subjected to the treatment with 3 µM CGRP and 3 µM BIBN4096 in the presence of 3 µM CGRP (n = 8 per group). Abbreviations: phosphorylated SFKs at Y416 (pSFKs), BIBN4096 (BIBN).
